# Internal and external training load in men’s professional padel players: A positional comparison in the Premier Padel circuit

**DOI:** 10.5114/biolsport.2026.154149

**Published:** 2026-03-24

**Authors:** Álvaro Bustamante-Sánchez, Diego Muñoz, Sergio José Ibáñez, Adrián Escudero-Tena, Rafael Conde-Ripoll

**Affiliations:** 1 Universidad Europea de Madrid. Department of Sports Sciences. Faculty of Medicine, Health and Sports. Calle Tajo, s/n, 28670 Villaviciosa de Odón, Madrid, Spain; 2 Research Group in Optimization of Training and Sports Performance (GOERD), Faculty of Sport Sciences, University of Extremadura, Caceres, Spain

**Keywords:** Racket sports, Performance, Heart rate, Accelerations, Decelerations, Distance

## Abstract

Significant positional differences exist between left- and right-side players in professional padel, particularly in movement patterns and shot selection during competition. However, little is known about how these roles influence internal and external training load. As training is key to optimizing performance, monitoring both physiological and physical stress is essential. This study compared training load responses between left- and right-side professional male padel players in the Premier Padel circuit. Thirty-four top-60 right-handed players wore Polar Team Pro sensors during 15 training sessions. Internal load was assessed through heart rate (HR; time in zones 50–60%, 60–70%, 70–80%, 90–100% HRmax), while external load was evaluated from acceleration and deceleration zones (0.50–0.99, 1.00–1.99, 2.00–2.99, 3.00–50.00 m/s^2^), speed zones (3.00–5.99, 6.00–8.99, 9.00–11.99, 12.00–14.99, ≥ 15.00 km/h), and distance per minute. The results showed no significant differences (p < 0.05) and small effect sizes in external load, although internal load revealed small and medium effect-size differences: left-side players performed more continuous efforts, both in volume and intensity, compared to right-side players, who engaged in intermittent effort of higher intensity but with lower involvement. On average, left- vs. right-side players recorded mean HRs of 72.42 ± 5.83% vs. 74.20 ± 5.82% HRmax, spent 31.7% vs. 27.4% of training time in zone 3 and 23.0% vs. 29.7% in zone 4, covered 22.31 ± 4.43 vs. 22.26 ± 4.49 m/min, and performed 6.61 ± 1.44 vs. 6.78 ± 0.71 accelerations/min. These positional benchmarks may guide future research and inform position-specific conditioning programs in professional padel.

## INTRODUCTION

Padel has swiftly become one of the fastest-growing sports globally, boasting over 30 million participants across 140 countries [1]. This high-paced [2] doubles sport demands seamless player coordination and strategic opposition [3, 4]. Played on a 20 x 10 meter court enclosed by glass walls and metal mesh, it offers a distinctive competitive environment [5]. With its rapid international expansion, multiple professional circuits have emerged, with Premier Padel now standing as the leading global tour.

Scientific interest in padel has grown substantially in recent years [6–9]. While much of this research has focused on performance analysis [10], particularly examining the game actions of professional players [11, 12], an equally crucial aspect remains less explored: the training load players endure to sustain and enhance their performance. Understanding these demands is essential for optimizing preparation and reducing injury risk [13, 14].

Training and match load refer to the cumulative stress athletes experience during training and competition, typically classified into internal (physiological responses) and external (physical workload) components [15]. In padel, several studies have examined internal load parameters—such as heart rate and ratings of perceived exertion—to assess the sport’s physiological demands [16–20]. However, despite these contributions, a critical gap remains: how internal load varies based on player position has yet to be thoroughly investigated.

On the other hand, external load, which reflects the physical demands athletes encounter, has been extensively studied in padel. Research in this area has primarily been categorized into four major domains: (1) analyses of temporal structure, including playing time and rest intervals [21]; (2) assessments of player movement patterns [22]; (3) evaluations of game scores [23]; and (4) investigations into technical-tactical actions [24]. Regarding positional differences in men’s padel, studies indicate that left-side and right-side players exhibit distinct shot-making tendencies throughout a match [25]. Leftside players execute more cross-court shots during rallies [26], and are more frequently involved in the second-to-last and final shots of each point [27]. Notably, they also hit more winners, reinforcing their reputation as the more aggressive players [25, 26]. From a movement perspective, left-side players perform a higher number of accelerations and decelerations per hour, further distinguishing their physical demands from their right-side counterparts [28]. These positional differences have also been confirmed through recent physical performance testing, with left-side players showing higher muscle mass and strength in sport-specific actions, while right-side players excelled in vertical jump tests [29]. All combined, these technical-tactical, and physical asymmetries suggest that left-side players may experience greater cumulative load during training, while right-side players may be exposed to more intermittent high-intensity efforts. This justifies the investigation of position-specific internal and external load profiles in professional padel.

Beyond the physiological, physical, technical, and tactical differences, playing position also influences psychological characteristics. Left-side players, for instance, tend to exhibit higher levels of somatic anxiety and self-confidence before competition compared to pressure training matches [30]. These psychological differences can, in turn, impact how players respond to training, making it even more crucial to monitor the training load’s effects on both well-being and performance [31, 32]. Despite the growing professionalization of padel, however, there is still limited understanding of the internal and external load demands placed on Premier Padel players during training sessions. Given that emotional states such as anxiety and confidence can influence autonomic nervous system activity, these psychological traits may partly explain individual or positional variation in heart rate responses or perceived training load.

Establishing reference values for internal and external training load is essential for evidence-based monitoring in elite padel. These normative data can serve as practical benchmarks to help coaches assess whether players are meeting expected load profiles based on their position. They may also support return-to-play decisions by identifying deviations from typical physiological and mechanical demands. Moreover, as the sport evolves, tracking these values over time can inform longitudinal comparisons of how training practices and physical requirements adapt at the highest levels of play.

Therefore, the aim of this study was to analyze and compare the training load responses of left-side and right-side male professional padel players, to provide insights into position-specific demands and their implications for training optimization. Based on previous research suggesting that left-side players exhibit higher match involvement, perform more accelerations, and cover greater distances [25–28], we hypothesized that they would also experience higher internal and external load metrics during training sessions compared to right-side players.

## MATERIALS AND METHODS

### Participants

Thirty-four top-60 male professional padel players of the Premier Padel circuit were analyzed. The players were divided in 2 groups according to their playing position: 19 left-side (height: 183.6 ± 1.61 cm; weight: 80.7 ± 3.07) and 15 right-side (height: 177.6 ± 3.94 cm; weight: 75.6 ± 4.84). Every single player was right-handed, eliminating further investigation bias. Before starting the research, the experimental procedures were explained to all the participants, who gave their voluntary written informed consent in accordance with the Declaration of Helsinki. The procedures conducted in the present research were designed and approved by the Ethical Committee of the University (CIPI/22.303).

### Procedure

We analyzed 15 training sessions (duration: 90.9 ± 5.20 minutes) in which we monitored training load through the following parameters of intensity: heart rate, accelerations, decelerations, and speed. The training sessions took place in the morning (from 9:30 to 12:30), from 3 to 1 weeks prior to the start of the competition. Each session followed a similar structure which consisted of a warm-up and session briefing, followed by three main training blocks:

– Warm-up (5 minutes): crosscourt, volley-baseline, and overhead parallel rallies (forehand and backhand), and session briefing.
– Technical-Tactical development (25 minutes). On-court coach-led drills focused on:
  - Under-pressure defensive wall returns
  - Defense-to-attack transitions
  - Net-control drills
  - Lob-overhead shots-volley-smash transitions
– Situational play (5 minute break and briefing + 25 minutes). Conditioned games, including:
  - Point-play initiated by the coach, developing different scenarios (e.g. corner-defence start)
  - Mini-sets with tactical constraints (e.g. points won only after a deep lob)
– Match simulation (5 minute break and briefing + 25 minutes): Real-game situations with scoreboard or time pressure.
  - Tie-break with time constraints (e.g. pair with higher ranking must win the tie-break in less than 6 minutes)
  - Handicap situations (e.g. servers start the game with 0–30 scoreboard)
  - Real-game situation.

### Instrumentation and study variables

**FIG. 1:**
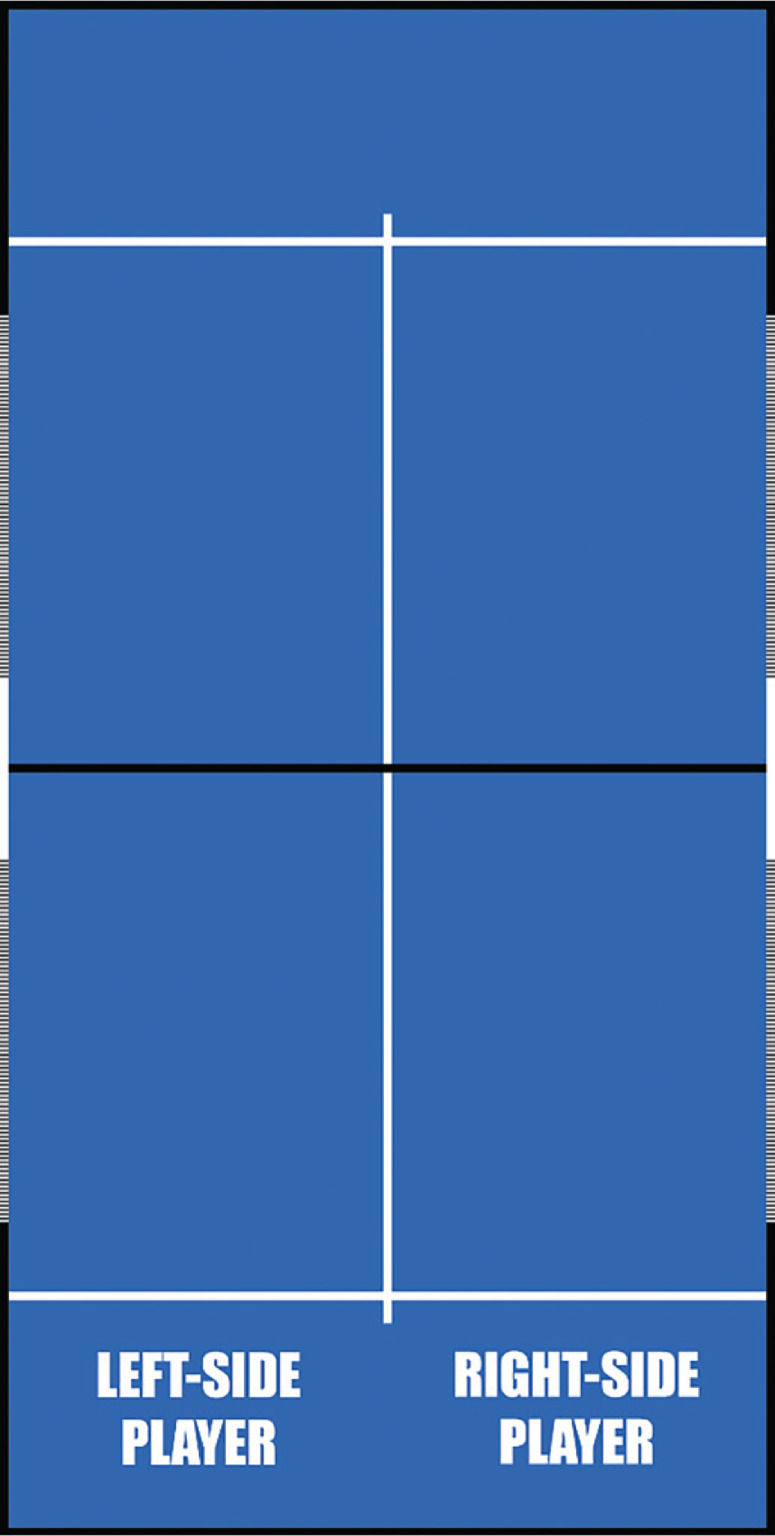
Side of play.

Figure 1 shows the independent variable: the side of play of the player, that is, the right-side or left-side.

All participants wore a motion sensor with a heart rate monitor, accelerometer, and gyroscope. Internal and external load were assessed through Polar Team Pro sensors and software (Polar Electro Oy, Finland), using the predefined thresholds by the Polar Team Pro system for intermittent court-based sports and following procedures of previous studies in padel [28, 33].

Specifically, we analyzed the following internal load variables:

– Heart rate (HR): maximum [bpm] (estimated by the formula maximum HR = 208 – (0.7 × age)) [34], average [bpm], time in zone 1 (50–60% of maximum HR) [min], time in zone 2 (60–70%) [min], time in zone 3 (70–80%) [min], time in zone 4 (80–90%) [min], time in zone 5 (90–100%) [min]. The time interval used to register heart rate data was 1-second, as specified in the Polar Team Pro device specifications (Polar Electro Oy, Finland).

Specifically, we analyzed the following external load variables:

– Accelerations: number of accelerations in zone 1 (0.50 to 0.99 m/s^2^), number of accelerations in zone 2 (1.00 to 1.99 m/s^2^), number of accelerations in zone 3 (2.00 to 2.99 m/s^2^), and number of accelerations in zone 4 (3.00 to 50.00 m/s^2^).
– Decelerations: number of decelerations in zone 1 (-0.99 to -0.50 m/s^2^), number of decelerations in zone 2 (-1.99 to -1.00 m/s^2^), number of decelerations in zone 3 (-2.99 to -2.00 m/s^2^), and number of decelerations in zone 4 (-50.00 to -3.00 m/s^2^).
– Speed: average speed [km/h], distance in the speed zone 1 [m] (3.00–5.99 km/h), distance in the speed zone 2 [m] (6.00–8.99 km/h), distance in the speed zone 3 [m] (9.00–11.99 km/h), distance in the speed zone 4 [m] (12.00–14.99 km/h), and distance the speed zone 5 [m] (≥ 15.00 km/h)
– Distance: total distance [m], and distance per minute [m/min]. In wearable devices, average speed is total distance divided by total time, while distance per minute (relative distance) is a more nuanced metric that indicates intensity or work rate per minute. While average speed includes any time the device registers as “stopped” or resting, relative distance focuses on the average distance covered in moving minutes, providing insight into an athlete’s average work rate, though both metrics have limitations and are influenced by tracking accuracy.

### Statistical analysis

Normality assumptions were tested using the Shapiro-Wilk test. Homoscedasticity assumptions were checked with the Levene test. Descriptive statistics were presented as mean and standard deviation. Differences between left-side and right-side players groups were analysed with a T-test. The Effect Size (ES) was tested by Cohen’s D, and interpreted as small for < 0.5 absolute values, medium for 0.5–0.8 absolute values and large for absolute values greater than 0.8. Jamovi 2.5.4 for Windows (Jamovi Project, Sydney, Australia) and SPSS (version 26.0; SPSS, Inc. Chicago, Illinois) were used. The level of significance for all the comparisons was set at p < 0.05.

## RESULTS

**FIG. 2:**
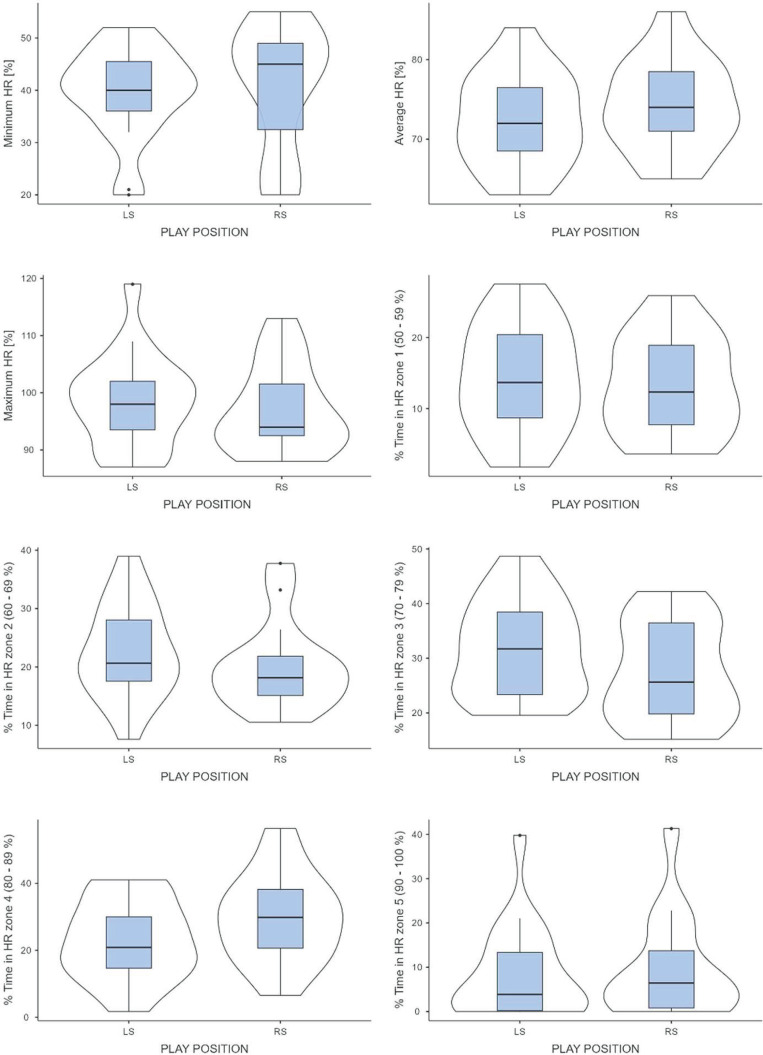
Internal load: heart rate variables. Data represents average values per player across all recorded training sessions. Note: RS: Right-side. LS: Left-side.

Figure 2 shows box and violin plots for internal load in left-side and right-side players. The rationale for using violin plots is based on the comprehensive visualization they provide of the distribution, density and central tendency of the data, which is particularly effective when comparing internal and external load variables across playing positions. This visualization enhances the interpretability of between-group and within-group variability required by this study. There were no significant differences (p < 0.05) between groups. We found higher values for left-side players (with a medium effect size) in the percentage of time in the zone 3 (70–80%) (31.72 ± 8.68 vs 27.38 ± 8.99, ES = 0.500), and lower values for left-side players in the percentage of time in zone 4 (80–90%) (23.03 ± 11.68 vs 29.69 ± 13.38, ES = -0.535).

We found a small effect size in the percentage of time in the zone 1 (50–60%) (14.23 ± 7.66 vs 13.18 ± 6.89, ES = 0.142), zone 2 (60–70%) (22.61 ± 7.99 vs 20.14 ± 7.53, ES = 0.316), and zone 5 (90–100%) (8.38 ± 10.42 vs 9.58 ± 6.42, ES = -0.109).

Similarly, there was a small effect size for the maximum heart rate (98.57 ± 8.05 vs 97.06 ± 7.68, ES = 0.191), the percentage of average heart rate (72.42 ± 5.83 vs 74.20 ± 5.82, ES = -0.305), and the percentage of minimum heart rate (39.52 ± 8.73 vs 40.73 ± 12.15, ES = -0.116).

Additionally, Table 1 shows heart-rate intensity zones for left-side and right-side players.

**TABLE 1:** Internal load data for left-side and right-side players.

	Left-side players		Right-side players		Student’s t	*p*	Cohen’s d

	M	M	SD	SD			
% time zone 1 (50–60%)	14.23	7.66	13.18	6.89	0.414	0.682	0.142
% time zone 2 (60–70%)	22.61	7.99	20.14	7.53	0.917	0.366	0.316
% time zone 3 (70–80%)	31.72	8.68	27.38	8.99	1.424	0.164	0.500
% time zone 4 (80–90%)	23.03	11.68	29.69	13.38	−1.548	0.131	−0.535
% time zone 5 (90–100%)	8.38	3.85	9.58	11.59	−0.317	0.753	−0.110

Note. M: mean. SD: Standard Deviation.

**FIG. 3:**
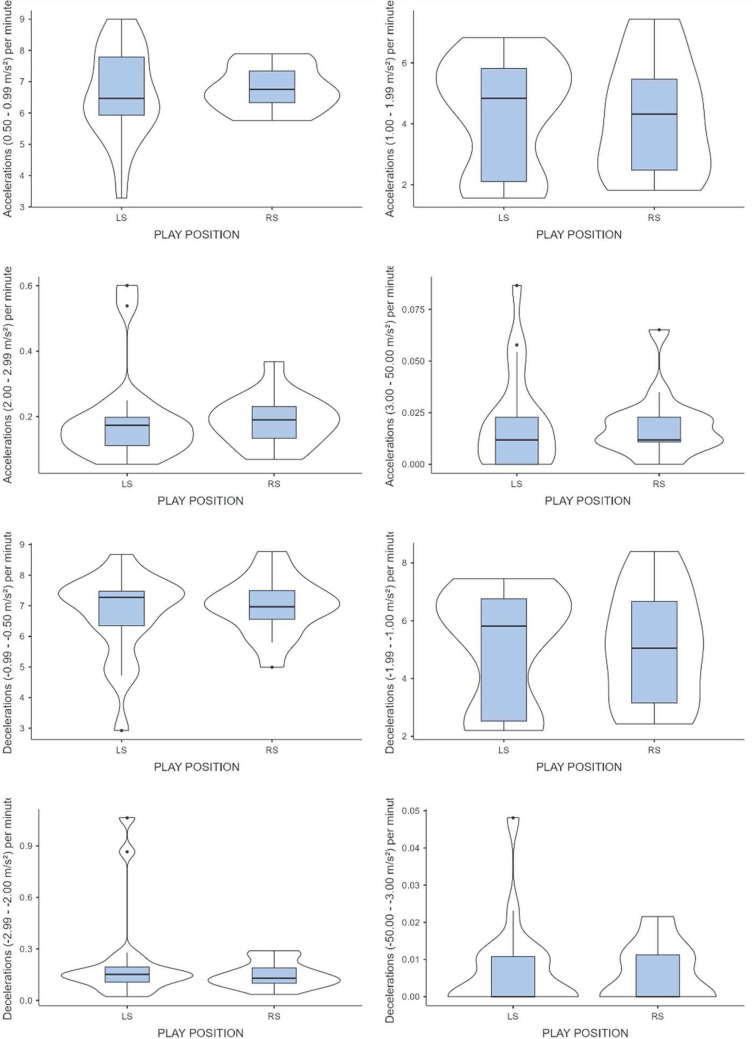
External load: accelerations and decelerations. Data represents average values per player across all recorded training sessions. Note: RS: Right-side. LS: Left-side.

Figure 3 shows box and violin plots for external load (accelerations and decelerations) in left-side and right-side players. There were no significant differences (p < 0.05) between groups.

We found similar values for left-side and right side players in the number of accelerations per minute in zone 1 (0.50 to 0.99 m/s^2^) (6.61 ± 1.44 vs 6.78 ± 0.71, ES = -0.137), accelerations per minute in zone 2 (1.00 to 1.99 m/s^2^) (4.33 ± 1.85 vs 4.18 ± 1.91, ES = 0.077), accelerations per minute in zone 3 (2.00 to 2.99 m/s^2^) (0.192 ± 0.144 vs 0.187 ± 0.078, ES = 0.044), and accelerations per minute in zone 4 (3.00 to 50.00 m/s^2^) (0.020 ± 0.023 vs 0.018 ± 0.016, ES = 0.104). The effect size was small for all the acceleration zones.

Similarly, no differences were found for the number of decelerations per minute in zone 1 (-0.99 to -0.50 m/s^2^) (6.69 ± 1.38 vs 6.99 ± 0.96, ES = -0.244), decelerations per minute in zone 2 (-1.99 to -1.00 m/s^2^) (5.11 ± 2.00 vs 5.08 ± 1.98, ES = 0.011), decelerations per minute in zone 3 (-2.99 to -2.00 m/s^2^) (0.226 ± 0.269 vs 0.151 ± 0.077, ES = 0.363), and decelerations per minute in zone 4 (-50.00 to -3.00 m/s^2^) (0.007 ± 0.012 vs 0.006 ± 0.007, ES = 0.059). The effect size was small for all the deceleration zones.

**FIG. 4:**
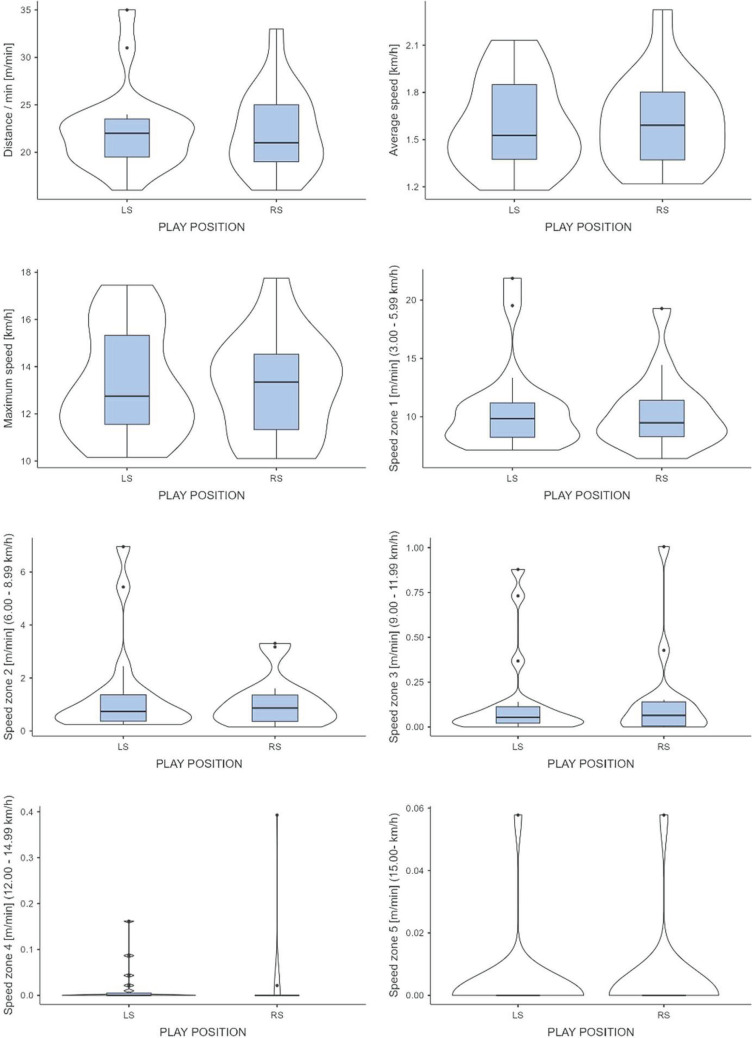
External load: speed and distances. Data represents average values per player across all recorded training sessions. Note: RS: Right-side. LS: Left-side.

Figure 4 shows box and violin plots for external load (speed and distances) in left-side and right-side players. There were no significant differences (p < 0.05) between groups.

We found similar values for left-side and right-side players in the distance per minute [m/min] (22.31 ± 4.43 vs 22.26 ± 4.49, ES = 0.011), average speed [km/h] (1.60 ± 0.288 vs 1.61 ± 0.295, ES = -0.040), maximum speed [km/h] (13.29 ± 2.30 vs 13.18 ± 2.18, ES = 0.046), meters per minute run in the speed zone 1 (3.00–5.99 km/h) (10.72 ± 3.90 vs 10.41 ± 3.20, ES = 0.083), in the number of meters per minute run in the speed zone 2 (6.00–8.99 km/h) (1.38 ± 1.80 vs 1.09 ± 0.976, ES = 0.191), in the number of meters per minute run in the speed zone 3 (9.00–11.99 km/h) (0.144 ± 0.248 vs 0.146 ± 0.262, ES = -0.009), in the number of meters per minute run in the speed zone 4 (12.00–14.99 km/h) (0.017 ± 0.041 vs 0.027 ± 0.101, ES = -0.143), and in the number of meters per minute run in speed zone 5 (15.00 or more km/h) (0.003 ± 0.013 vs 0.004 ± 0.015, ES = -0.057). The effect size was small for all the effect sizes studied.

## DISCUSSION

The aim of this study was to analyze and compare the training load responses of left-side and right-side male professional padel players, providing insights into position-specific demands and their implications for training optimization.

The findings of this study show values of mean heart rate (HR) similar to previous studies in padel players, with a mean of 75–85% of maximum HR registered in professional padel players during simulated competition [17, 35, 36]. We can observe no significant differences with small and medium effect sizes between right- and leftside players attending to heart rate. As shown by the results, players on the right side perform more intermittent efforts, with higher average HR percentages, specifically a greater percentage of time spent in zone 4. However, left-side players maintain similar percentages in zones 2, 3, and 4, which could indicate a more continuous submaximal effort. This fact could be relevant in order to design training sessions for left- and right-side players. Additionally, the medium effect-size obtained in favour of left-side players in zone 3, may indicate their sustained involvement in rally phases, whereas the medium effect-size favouring right-side players in zone 4 may reflect more intermittent, high-intensity demands associated with defensive wall returns under pressure. Previous studies reported a higher involvement of left-side players in the game [27, 37], being the most involved in the finishing actions of the points [25, 38], performing a higher percentage of trays, smashes, side-wall, and wall boast shots than the players on the right side. In addition, it has been reported that left-side players of the qualifying draw make more unforced errors and fewer winners than those of the main draw [39], highlighting the importance of physical fitness in high levels of play, especially those players positioned on the left side, who exhibit a higher level of involvement in the game.

Generally, the game patterns of pairs tend to seek situations that favor the involvement of players with greater finishing ability, with the participation of right-side players being more intermittent. In this sense, the most significant variations in HR of these players obtained in this study could confirm this fact. A recent study affirmed that leftside players face greater physical demands due to technical-tactical actions requiring more court coverage [28]. These same results were reported previously by Fernandez de Osso [38], observing that the zones with the higher percentages of strokes were those of the leftside player.

From a training perspective, the observed differences in heart rate zone distribution suggest that left-side players may benefit from conditioning that emphasizes aerobic capacity and tolerance to sustained submaximal effort, reflecting their continuous involvement in rallies. In contrast, the higher percentage of time that right-side players spend in zone 4 indicates a more intermittent physiological profile, with repeated bursts of high intensity interspersed with recovery. These distinctions may guide individualized conditioning programs, with left-side players focusing more on aerobic endurance and prolonged work intervals, and right-side players incorporating more high-intensity interval training (HIIT) to reflect their match-specific intensity demands.

Finally, reduced percentages of time were obtained in zone 5 on both sides of play, which could be related to the characteristics of the game. Although the intensity of the game is high, as shown in studies where the hitting rate is 0.80 shots per second [21], the duration of the points is low. Previous studies report that more than 60% of the points last less than 10 seconds [40, 41]. To date, no studies have found high concentrations of lactate in professional padel players. Regarding male players in the national category, their values range between 2.87 ± 1.48 mmol/L and 3.38 ± 1.83 mmol/L depending on the level of play [42]. The overall metabolic profile of a padel match must also consider the cumulative session duration and the sustained cardiovascular response, which suggests a significant contribution of the aerobic system throughout the match [43].

The results obtained in internal load parameters are also confirmed by external load parameters, such as accelerations and decelerations. Right-side players exhibit greater variability in their accelerations, whereas left-side players perform a higher number of submaximal intensity accelerations per minute (1.00–1.99 m/s^2^). One of the relevant aspects is that 90% of the accelerations and decelerations occur between 0.50–1.99 m/s^2^ in all players, which could be related to the short distances covered during the game. Miralles et al [28] obtained similar results, observing that approximately 80% of all accelerations occurred within the 1–2 meter range. In addition, these authors reported that players on the left side performed a significantly higher number of accelerations per hour compared to those on the right side. These results provide specific parameters for professional padel training.

Finally, our findings show that left-side players cover a greater distance at a lower speed, which could indicate more continuous participation compared to right-side players. Additionally, left-side players execute shots across a greater percentage of the court (such as the central zone), especially when positioned near the net and in the transition zone [25], thus covering a greater distance during the points. Previously, Ramón-Llin [44] found that players on the left side, covered significantly more distance than their partners, when both players were right-handed [44].

Taken together, the combined internal and external load data confirm that left-side players sustain more continuous workloads, while right-side players engage in shorter, higher-intensity efforts. These findings support the development of position-specific monitoring and training frameworks in padel. For example, coaches may consider adapting work-rest ratios, load progression, and tactical drills to reflect the differing physiological and movement profiles associated with each playing side. Implementing load monitoring by playing position may enhance individualized performance planning and reduce the risk of undertraining or overtraining specific athletes. Additionally, left-side players exhibited greater variability in several key load metrics, including HR zone 5, acceleration zones 1, 3, and 4, deceleration zones 1, 3, and 4, and speed zone 2. This variability may reflect the wider range of physical responses required due to their more continuous engagement in rallies, especially on those balls that they must take in the middle zone of the court. In contrast, rightside players demonstrated greater variability in speed zones 4 and 5, possibly indicating intermittent bursts of high-speed movements associated with reactive or transitional play. These patterns may have practical implications for individualized training adaptations and load management strategies.

The results obtained in this study have some limitations that should be considered when interpreting the findings. For example, the different roles of each player (server, returner, server partner and returner partner) were not taken into account, which may affect external load parameters, particularly the distance covered, as reported by Ramón-Llin et al [45], where the server is the player who covers the most distance during a point. To better reflect the real-training context of elite padel players, this research observed practice sessions with no intervention in the coach’s drill design to ensure an ecological perspective. This is both a strength and a limitation of the study. The observation and monitoring of real training scenarios contributed to the development of a training-session structure template explained in the procedure subsection together with the internal and external load differences by playing position in elite padel players. However, the inner different training sessions’ content variability, and the absence of competitive matches analysis are some of the limitations of the study. With the aim of ensuring consistency and facilitating comparisons across similar intermittent sports, we opted to reuse predefined threshold zones for court-based intermittent sports. Although it is out of the scope of this study, future research could consider these results to develop ad-hoc thresholds for quantifying speeds and accelerations in padel. On the other hand, the match outcome could be a factor to consider for future research, as the winning pairs, which are typically those that spend more time at the net, could cover different distances and at different intensities compared to the losing pairs. Finally, future research should quantify both external and internal load during competitive matches, together with their correlation matrix, to develop a specific methodology according to the real game demands.

## CONCLUSIONS

This study presents new insights into the training workload of professional padel players based on the playing side. Left-side players were found to engage in more continuous efforts, with higher volume and submaximal intensity, while right-side players exhibited more intermittent but higher-intensity exertion with lower overall involvement. These findings suggest that professional padel players experience different physical and physiological demands depending on their positional role. Accordingly, training prescriptions and load management strategies should be tailored by playing side to better reflect the specific effort profiles of each position. This position-specific approach could improve training efficiency, reduce injury risk, and enhance performance. Future research is encouraged to build on these findings using larger samples and competition-based data.

## Data Availability

Data will be available upon request.
